# Twenty‐year study of in‐hospital and postdischarge mortality following emergency general surgical admission

**DOI:** 10.1002/bjs5.50187

**Published:** 2019-07-09

**Authors:** G. Ramsay, J. M. Wohlgemut, J. O. Jansen

**Affiliations:** ^1^ Rowett Institute; ^2^ School of Medicine, Medical Sciences and Nutrition University of Aberdeen Aberdeen; ^3^ Department of General Surgery Raigmore Hospital Inverness; ^4^ Department of General Surgery Inverclyde Royal Hospital Greenock UK; ^5^ Division of Acute Care Surgery University of Alabama at Birmingham Birmingham Alabama USA

## Abstract

**Background:**

Emergency general surgery (EGS) patients have a higher mortality than those having elective surgery. Few studies have investigated changes in EGS‐associated mortality over time or explored mortality rates after discharge. The aim of this study was to conduct a comprehensive, population‐based analysis of mortality in EGS patients over a 20‐year time frame.

**Methods:**

This was a cross‐sectional study of all adult EGS admissions in Scotland between 1996 and 2015. Data were obtained from national records. Co‐morbidities were defined by Charlson Co‐morbidity Index, and operations were coded by OPCS‐4 classifications. Linear and multivariable logistic regression models were used to evaluate changes over time.

**Results:**

Among 1 450 296 patients, the overall inpatient, 30‐day, 90‐day and 1‐year mortality rates were 1·8, 3·8, 6·4 and 12·5 per cent respectively. Mortality was influenced by age at admission, co‐morbidity, operation performed and date of admission (all *P* < 0·001), and improved with time on subgroup analysis by age, co‐morbidity and operation status. Medium‐term mortality was high: the 1‐year mortality rate in patients aged over 75 years was 35·6 per cent. The 1‐year mortality rate in highly co‐morbid patients decreased from 75·1 to 57·1 per cent over the time frame of the study (*P* < 0·001).

**Conclusion:**

Mortality after EGS in Scotland has reduced significantly over the past 20 years. This analysis of medium‐term mortality after EGS admission demonstrates strikingly high rates, and postdischarge death rates are higher than is currently appreciated.

## Abstract (es)

**Antecedentes:**

Los pacientes sometidos a cirugía general urgente (*emergency general surgery*, EGS) presentan una mortalidad más elevada que los pacientes sometidos a cirugía electiva. Pocos estudios han investigado los cambios en la mortalidad asociada a la EGS a lo largo del tiempo o han analizado las tasas de mortalidad tras el alta hospitalaria. El objetivo de este estudio fue llevar a cabo un análisis exhaustivo de base poblacional de la mortalidad en pacientes en EGS durante un horizonte temporal de 20 años.

**Métodos:**

Se trata de un estudio transversal de todos los ingresos de adultos por EGS en Escocia entre 1996 y 2015. Los datos se obtuvieron de los registros nacionales. Las comorbilidades se definieron según el índice de comorbilidad de Charlson y las operaciones se codificaron con las clasificaciones OPCS4. Se utilizaron modelos de regresión logística lineal y multivariante para evaluar cambios a los largo del tiempo.

**Resultados:**

En un total de 1.450.296 pacientes, las tasas globales de mortalidad hospitalaria, a los 30 días, 90 días y un año fueron de 1,8%, 3,8%, 6,4% y 12,5%, respectivamente. La mortalidad estaba influida por la edad en el momento del ingreso, comorbilidad, la intervención quirúrgica realizada y la fecha de ingreso (todas las variables *P* < 0,001) y mejoró con el tiempo en el análisis por subgrupos de edad, comorbilidad e intervenciones quirúrgicas. La mortalidad a medio plazo fue elevada: la tasa de mortalidad a un año en pacientes mayores de 75 años fue de 35,6%. La mortalidad a un año en pacientes con elevada morbilidad disminuyó de un 75% a un 57% a lo largo del periodo del estudio (*P* < 0,001).

**Conclusión:**

La mortalidad tras EGS en Escocia ha disminuido significativamente a lo largo de los últimos 20 años. No obstante, el análisis de la mortalidad a medio plazo después de un ingreso por EGS demuestra unas tasas sorprendentemente elevadas y, por ello, actualmente hay que tener en cuenta la mortalidad tras el alta de forma especial.

## Introduction

Emergency general surgery (EGS) is the unscheduled in‐hospital treatment of patients under the care of a surgeon with training in gastrointestinal surgery. EGS is a key component of the specialty of general surgery, with an estimated 50 per cent of inpatient general surgical beds in the UK being used to accommodate such patients^1^. In the USA, EGS accounts for 3 million hospitalizations per year, or 7 per cent of the total^2^, ^3^.

EGS patients have a high risk of dying^4^, ^5^, ^6^ – up to eight times that of patients admitted electively^4^, ^7^. However, few studies have investigated changes in EGS‐associated mortality over time. A previous report^8^ on secular trends in diagnosis, types of operation and age/sex‐adjusted in‐hospital mortality over a 20‐year period has been published recently, and showed an increasing number of admissions of elderly patients, with a constant number of operations. As part of this study, trends in inpatient mortality demonstrated a decrease in the number of inpatient deaths over time, standardized for age and sex^8^. However, there is a substantial burden of mortality after discharge from acute care, especially in older patients^9^, and trends in postdischarge mortality are yet to be established.

There have been many analyses of specific surgical conditions^10^ and subpopulations^11^, ^12^, as well as a variety of mortality prediction models^13^, but most of these studies focused on inpatient or early postoperative mortality. Mortality rates beyond discharge and standard outcome time frames such as 30 days are rarely reported. Moreover, most studies, including current initiatives such as the National Emergency Laparotomy Audit in the UK, are limited to patients undergoing operative treatment^14^, ^15^, ^16^, ^17^, whereas only 10–28 per cent of patients admitted under the care of a general surgeon have an operation^8^, ^9^, ^18^.

The present study aimed to evaluate the secular trends in mortality after EGS admission with particular reference to postdischarge mortality, operative compared with non‐operative treatment, and the impact of co‐morbidity.

## Methods

This was a population‐based, cross‐sectional study, set in Scotland, and a further analysis of data used in a previous publication^8^. For the present analysis, medium‐term mortality data were obtained. The National Health Service (NHS) in Scotland collects data on all hospital admissions, through its Information Services Division (ISD). Patients are assigned a unique Community Health Index number at their first contact with NHS Scotland services^19^. ISD data are linked to death records; this allows mortality to be analysed even once patients have been discharged from the acute care setting, or if they are admitted to another facility, and enables a comprehensive, population‐based evaluation of outcome and service.

This project was approved by the Public Benefit and Privacy Panel of NHS Scotland (reference 1617‐0207) and registered with the research governance department of NHS Grampian and the University of Aberdeen.

### Data sources

The ISD uses a nationally agreed coding mechanism to ensure accuracy and consistency. Data are coded locally, and then stored centrally. Individualized patient data, anonymized at source, were used. These were transferred to the Data Safehaven of the University of Aberdeen for analysis. Demographic details (age and sex), co‐morbidity profile, date and type of admission (transfer from ward or admission from home), and date of death (if applicable) were available for every patient. Admission diagnoses were available in the form of ICD‐10 codes. Operative details were coded using OPCS4. Operations were included if they had an OPCS4 code with a G–J (upper gastrointestinal tract, lower gastrointestinal tract, other abdominal organ, principally digestive) or T (soft tissue) prefix.

### Case definition and terminology

An EGS patient was defined as a patient aged 16 years or more, admitted to a Scottish hospital, under the care of a consultant general surgeon (specialty code C1), either directly from the community or from another hospital ward or hospital, as an emergency (also sometimes referred to as unscheduled or unplanned admissions), between 1 April 1996 and 31 December 2015.

### Statistical analysis

Inpatient mortality (defined as death during the index admission) and mortality at 30 days, 90 days and 1 year after the day of admission were analysed. To ensure adequate follow‐up, data captured were limited to index admissions from 1996 to 2015. Annual trends were calculated from 1997 onwards due to incomplete capture of data in 1996. Patients were ‘virtually followed up’ until 1 January 2017, ensuring a minimum follow‐up of 1 year for all subjects. Rather than present age‐ and sex‐adjusted mortality, which are often difficult to comprehend, the results were stratified by age group: 16–30, 31–45, 46–60, 61–75 and more than 75 years. Mortality at all time points was analysed in subgroups of whether admitted patients underwent an operation or not, and by degree of co‐morbidity. Co‐morbidity was quantified using the Charlson Co‐morbidity Index (CCI), with 10‐year lookback^20^, and divided into three groups: no co‐morbidity (CCI score 0), co‐morbidity (CCI score 1–4) and severe co‐morbidity (CCI score above 4).

Univariable linear regression was used to analyse changes in mortality rates over time. A multivariable logistic regression model, adjusting for age, operative status, sex and co‐morbidity (by CCI group) was then created^20^. Data were analysed using a combination of Microsoft Excel® v16.0 (Microsoft, Redwood, Washington, USA) and SPSS® v24.0 (IBM, Armonk, New York, USA). Categorical data were analysed with χ^2^ tests, and ordinal data using Mann–Whitney *U* tests.

## Results

Between 1996 and 2015, there were 1 450 296 emergency admissions, of 865 146 patients, under the care of a general surgeon in Scotland. Of these admissions, 696 901 (48·1 per cent) involved male and 753 388 (51·9 per cent) female patients; sex was not known for seven patients. Over the 20‐year study period, there was a median of 72 145 admissions per year. The annual number of admissions increased over time from 65 033 in 1997 to 79 333 in 2015 (*P* < 0·001), as reported previously^8^. A total of 395 019 admissions resulted in operative intervention (27·2 per cent), with a median of 20 089 operations per year. In contrast to the number of admissions, there was no increase in the number of operations per year. The median age of patients increased from 52 (i.q.r. 33–71) in 1996 to 53 (35–70) in 2015. The proportion of patients with severe co‐morbidity (CCI score above 4) remained stable over the duration of the study (24 347 patients, 1·7 per cent). In contrast, the proportion of patients with mild/moderate co‐morbidity (CCI grade 1–4) decreased over time (*P* = 0·016), and the proportion with no co‐morbidity increased (*P* < 0·001) (*Table* 1).

**Table 1:** Demographics of cohort by year of study time frame

Year	No. of admissions	No. of operations*	No. of male patients*	No. of female patients*	Median age (years)†	Significant co‐morbidity (CCI > 4)*	Moderate co‐morbidity (CCI 1–4)*	No co‐morbidity (CCI 0)*
1996	47 803	13 749 (28·8)	24 189 (50·6)	23 614 (49·4)	52 (33–71)	763 (1·6)	14 006 (29·3)	33 034 (69·1)
1997	65 033	19 073 (29·3)	32 773 (50·4)	32 260 (49·6)	52 (33–70)	1139 (1·8)	18 860 (29·0)	45 034 (69·2)
1998	65 232	19 352 (29·7)	32 751 (50·2)	32 481 (49·8)	52 (34–70)	1172 (1·8)	19 680 (30·2)	44 380 (68·0)
1999	65 817	19 398 (29·5)	33 270 (50·5)	32 547 (49·5)	51 (34–70)	1253 (1·9)	18 806 (28·6)	45 758 (69·5)
2000	69 774	19 936 (28·6)	35 235 (50·5)	34 538 (49·5)	52 (34–70)	1332 (1·9)	19 599 (28·1)	48 843 (70·0)
2001	71 977	20 358 (28·3)	36 150 (50·2)	35 827 (49·8)	52 (34–70)	1304 (1·8)	20 347 (28·3)	50 326 (69·9)
2002	71 823	20 042 (27·9)	36 355 (50·6)	35 468 (49·4)	52 (35–70)	1269 (1·8)	19 918 (27·7)	50 636 (70·5)
2003	71 278	20 470 (28·7)	35 100 (49·2)	36 178 (50·8)	53 (35–71)	1226 (1·7)	20 163 (28·3)	49 889 (70·0)
2004	72 313	20 961 (29·0)	35 536 (49·1)	36 777 (50·9)	53 (36–71)	1232 (1·7)	20 555 (28·4)	50 526 (69·9)
2005	70 909	20 687 (29·2)	34 326 (48·4)	36 583 (51·6)	53 (36–71)	1310 (1·8)	20 033 (28·3)	49 566 (69·9)
2006	71 073	20 770 (29·2)	34 118 (48·0)	36 955 (52·0)	53 (36–71)	1157 (1·6)	19 686 (27·7)	50 230 (70·7)
2007	73 585	20 994 (28·5)	35 508 (48·3)	38 077 (51·7)	53 (36–71)	1176 (1·6)	20 093 (27·3)	52 316 (71·1)
2008	76 278	21 454 (28·1)	36 515 (47·9)	39 763 (52·1)	52 (36–70)	1089 (1·4)	20 305 (26·6)	54 884 (72·0)
2009	77 861	21 022 (27·0)	36 879 (47·4)	40 980 (52·6)	52 (36–70)	1239 (1·6)	20 751 (26·7)	55 871 (71·8)
2010	76 756	20 136 (26·2)	35 914 (46·8)	40 840 (53·2)	53 (36–70)	1261 (1·6)	20 088 (26·2)	55 407 (72·2)
2011	79 453	20 323 (25·6)	36 940 (46·5)	42 513 (53·5)	52 (35–70)	1225 (1·5)	20 328 (25·6)	57 900 (72·9)
2012	81 568	19 607 (24·0)	37 067 (45·4)	44 500 (54·6)	52 (35–70)	1298 (1·6)	20 587 (25·2)	59 683 (73·2)
2013	81 475	19 650 (24·1)	36 610 (44·9)	44 865 (55·1)	52 (35–70)	1285 (1·6)	20 272 (24·9)	59 918 (73·5)
2014	80 955	19 174 (23·7)	36 215 (44·7)	44 740 (55·3)	53 (35–70)	1324 (1·6)	20 292 (25·1)	59 339 (73·3)
2015	79 333	17 863 (22·5)	35 450 (44·7)	43 882 (55·3)	53 (35–70)	1293 (1·6)	19 549 (24·6)	58 491 (73·7)

Values in parentheses are *percentages and †interquartile range.

### Change in mortality over time

The all‐cause inpatient mortality rate over the study period was 1·8 per cent, 30‐ and 90‐day mortality rates were 3·8 and 6·4 per cent respectively, and the 1‐year mortality rate was 12·5 per cent. All of these values decreased over time, although the rate of decrease was greater for later time points: mean reduction 0·099 per cent per annum for inpatient mortality, 0·119 per cent for 30‐day mortality, 0·167 per cent for 90‐day mortality, and 0·274 per cent for 1‐year mortality (all *P* < 0·001) (*Table* *S1*, supporting information).

### Mortality analysis by age group

**Figure 1:**
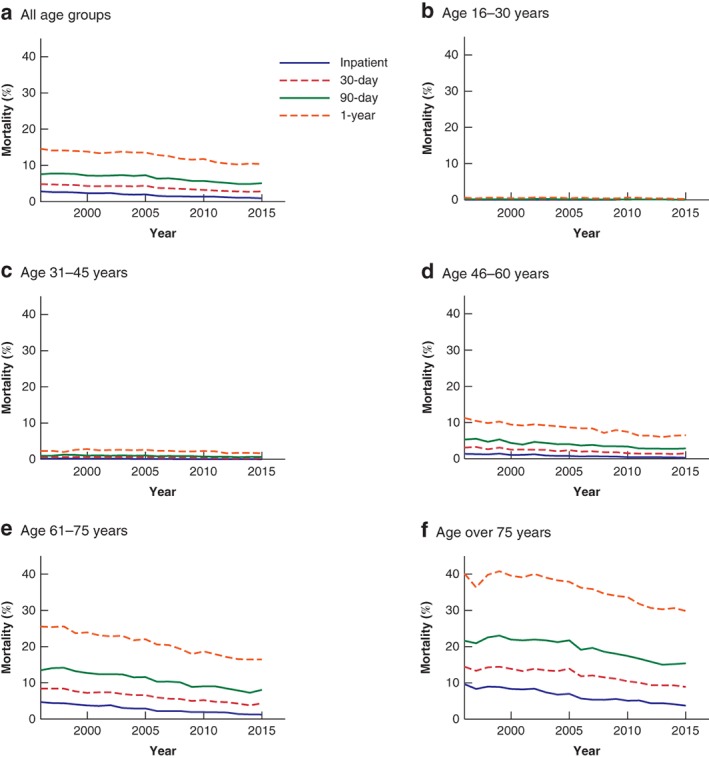
Mortality of emergency general surgery patients over time, stratified by age group **a** All age groups; **b** age 16–30 years; **c** age 31–45 years; **d** age 46–60 years; **e** age 61–75 years; **f** age over 75 years. Results of the linear regression analysis, including slope, 95 per cent c.i. and *P* values, are shown in *Table* *S2* (supporting information).

The mortality rate increased with age at all mortality time points studied (*P* < 0·001) (*Fig*. 1). The inpatient mortality rate was 0·03 per cent in 16–30‐year‐olds, 0·15 per cent in 31–45‐year‐olds, 0·8 per cent in 46–60‐year‐olds, 2·7 per cent in 61–75‐year‐olds and 6·3 per cent in those aged over 75 years. The 30‐day, 90‐day and 1‐year mortality rates were all higher, increasing to 35·6 per cent in patients aged over 75 years at 1 year. When analysed by age group, mortality decreased over time for each stratum, and at all four time points. Decreases were most marked in older patients, with over 75‐year‐olds experiencing the greatest reduction. The inpatient mortality rate in this group decreased by 0·307 per cent per year, 30‐day mortality by 0·312 per cent per year, 90‐day mortality by 0·429 per cent per year, and 1‐year mortality by 0·582 per cent per year (all *P* < 0·001). Similar changes were seen in younger patients, although they were less pronounced. In 16–30‐year‐olds, the inpatient mortality rate decreased by 0·002 per cent per year (*P* < 0·001), 30‐day mortality by 0·006 per cent per year (*P* < 0·001), 90‐day mortality by 0·007 per cent per year (*P* < 0·001) and 1‐year mortality by 0·012 per cent per year (*P* = 0·007). The 31–45‐year‐olds, 46–60‐year‐olds and 61–75‐year‐olds had reductions that lay between the two extremes of age (*Fig*. 1).

### Operative and non‐operative treatment

**Figure 2:**
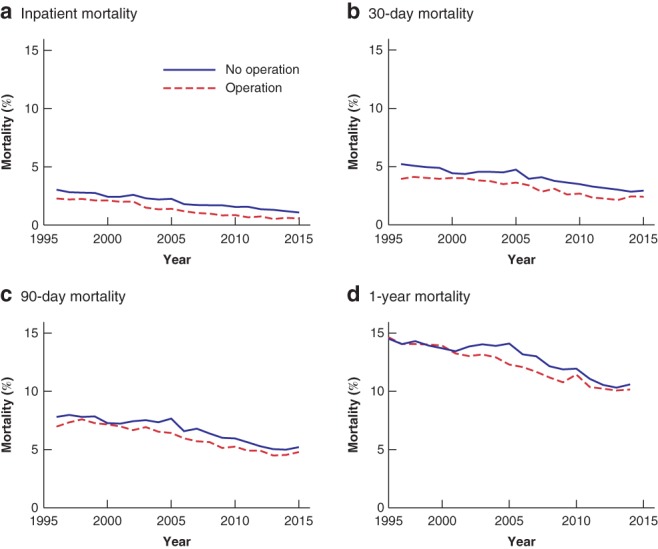
Mortality of emergency general surgery patients over time, by operative status during index admission **a** Inpatient mortality; **b** 30‐day mortality; **c** 90‐day mortality; **d** 1‐year mortality. Results of the linear regression analysis, including slope, 95 per cent c.i. and *P* values, are shown in *Table* *S3* (supporting information).

Overall inpatient, 30‐day, 90‐day and 1‐year mortality rates in patients who had an operation were 1·4, 3·3, 6·0 and 12·2 per cent respectively (*Fig*. 2). Patients who did not have an operation had slightly higher rates than those who had a procedure at all time points (1·9, 3·8, 6·2 and 12·6 respectively; all *P* < 0·001). Inpatient mortality in patients who underwent operative management decreased by 0·106 per cent per year (*P* < 0·001). Longer time points were again associated with a greater decrease: 0·115 per cent for 30‐day mortality, 0·171 per cent for 90‐day mortality, and 0·261 per cent for 1‐year mortality (all *P* < 0·001). For patients who did not have an operation during their emergency admission, mortality at different time points decreased similarly (all *P* < 0·001).

### Co‐morbidity

**Figure 3:**
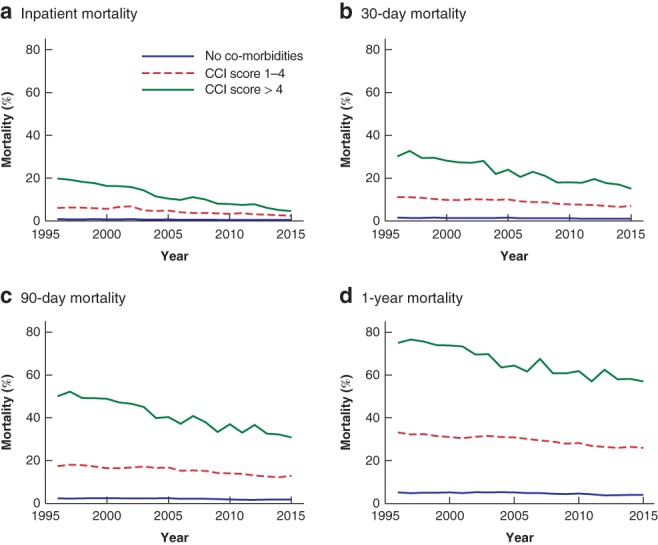
Mortality of emergency general surgery patients over time, by Charlson Co‐morbidity Index **a** Inpatient mortality; **b** 30‐day mortality; **c** 90‐day mortality; **d** 1‐year mortality. CCI, Charlson Co‐morbidity Index. Results of the linear regression, including slope, 95 per cent c.i. and *P* values, are shown in *Table* *S4* (supporting information).

Patients in the co‐morbidity (CCI score 1–4) and severe co‐morbidity (CCI score above 4) groups had higher mortality than those with no co‐morbidity (CCI score 0) at all four time points (*Fig*. 3). Mortality was related to extent of co‐morbidity: patients with no co‐morbidity had very low inpatient, 30‐day, 90‐day and 1‐year mortality. In contrast, those with co‐morbidity (CCI score 1–4) had an inpatient mortality rate of 4·4 per cent, 30‐day mortality rate of 9·0 per cent, 90‐day mortality rate of 15·4 per cent, and 1‐year mortality rate of 29·5 per cent, with annual improvements of 0·149, 0·237, 0·303 and 0·380 per cent respectively over time. At the beginning of the study, patients with severe co‐morbidity (CCI score above 4) had high inpatient (19·8 per cent), 30‐day (30·1 per cent), 90‐day (50·1 per cent) and 1‐year (75·1 per cent) mortality. However, these values improved markedly over time: over the duration of the study there was a 0·82 per cent reduction per year in hospital mortality, a 0·87 per cent per year reduction in 30‐day mortality, a 1·14 per cent per annum reduction in 90‐day mortality, and a 1·09 per cent per annum reduction in 1‐year mortality (all *P* < 0·001). At the end of the study, the 1‐year mortality rate for EGS patients with severe co‐morbidity had decreased from 75·1 to 57·1 per cent.

### Multivariable analysis

Patient age, operative intervention, underlying co‐morbidity and year of admission all influenced mortality at all four time points studied in this analysis. Furthermore, there were changes in patient characteristics and operative management over the 20 years of the study (*Table* 1). A multivariable model was therefore created to assess whether secular trends in mortality improvement were independent of these confounders (*Table* 2). At each mortality time point studied, year of admission was an independent factor for outcome. Sex, operative status, co‐morbidity and age were also independent predictors.

**Table 2:** Multivariable logistic regression model for in‐hospital, 30‐day, 90‐day and 1‐year mortality emergency general surgery index index admission

	In‐hospital mortality		30‐day mortality		90‐day mortality		1‐year mortality	
	Odds ratio	*P*	Odds ratio	*P*	Odds ratio	*P*	Odds ratio	*P*
**Time**								
Year of admission	0·95 (0·94, 0·95)	< 0·001	0·97 (0·97, 0·97)	< 0·001	0·97 (0·97, 0·97)	< 0·001	0·98 (0·98, 0·98)	< 0·001
**Sex**								
M	1·00 (reference)		1·00 (reference)		1·00 (reference)		1·00 (reference)	
F	1·11 (1·08, 1·14)	< 0·001	0·98 (0·96, 0·99)	< 0·001	0·95 (0·94, 0·97)	< 0·001	0·88 (0·087, 0·89)	< 0·001
**Operative status**								
No operation	1·00 (reference)		1·00 (reference)		1·00 (reference)		1·00 (reference)	
Operation	0·66 (0·64, 0·68)	< 0·001	0·80 (0·78, 0·81)	< 0·001	0·91 (0·90, 0·93)	< 0·001	0·97 (0·95, 0·98)	< 0·001
**Co‐morbidity**								
None	1·00 (reference)		1·00 (reference)		1·00 (reference)		1·00 (reference)	
CCI score 1–4	3·80 (3·68, 3·92)	< 0·001	3·72 (3·64, 3·80)	< 0·001	4·25 (4·18, 4·32)	< 0·001	4·59 (4·53, 4·64)	< 0·001
CCI score > 4	12·50 (11·90, 13·12)	< 0·001	12·92 (12·47, 13·39)	< 0·001	19·11 (18·57, 19·70)	< 0·001	27·42 (26·60, 28·25)	< 0·001
**Age group (years)**								
16–30	1·00 (reference)		1·00 (reference)		1·00 (reference)		1·00 (reference)	
31–45	3·40 (2·73, 4·24)	< 0·001	3·54 (3·14, 4·00)	< 0·001	3·73 (3·40, 4·09)	< 0·001	3·49 (3·30, 3·70)	< 0·001
46–60	13·03 (10·61, 16·00)	< 0·001	10·86 (9·70, 12·16)	< 0·001	11·23 (10·30, 12·24)	< 0·001	9·58 (9·08, 10·11)	< 0·001
61–75	34·64 (28·30, 42·40)	< 0·001	24·94 (22·30, 27·88)	< 0·001	25·12 (23·07, 27·36)	< 0·001	21·21 (20·11, 22·40)	< 0·001
> 75	83·77 (68·48, 102·49)	< 0·001	52·20 (46·68, 58·30)	< 0·001	51·24 (47·06, 55·79)	< 0·001	47·53 (45·10, 50·14)	< 0·001

Values in parentheses are 95 per cent confidence intervals. CCI, Charlson Co‐morbidity Index.

## Discussion

This population‐based, 20‐year analysis has demonstrated significant reductions in inpatient, 30‐day, 90‐day and 1‐year mortality following emergency general surgical admission. The secular reductions in mortality were observed across all subgroup analyses, by age, operative status and co‐morbidity, and were more marked at later time points. In addition, the improvements in mortality over time were independent of these factors on multivariable analysis.

Reductions in age‐ and sex‐standardized inpatient mortality rates of EGS patients have been reported previously^8^. The present study adds an in‐depth analysis of these trends, by examining mortality at later time points and following discharge from acute care. The improvements seen were particularly striking in those at high risk, such as the elderly, and those with extensive co‐morbidities, suggesting gradual improvements in the system of care. Improved mortality in patients having operations as well as those who were managed without surgery also suggests a more systemic improvement in clinical care, than merely a change in perioperative practice^21^, ^22^, ^23^, or better case selection. Improvements in critical care, and also increased liaison with family doctors and other services, such as geriatric and community specialties, multidisciplinary team working and more frequent use of radiological diagnostics, may all have contributed to these improvements in clinical outcomes. However, the reasons behind the mortality improvements seen in this study are beyond the scope of the present analysis. Further improvements could potentially be seen by increasing multidisciplinary links, particularly in the frail elderly cohorts, and adopting an enhanced recovery approach to patients who have an operation^24^.

The present study analysed outcomes in a longer follow‐up time frame than that usually assessed for EGS mortality studies^25^, ^26^, ^27^, ^28^. Despite the improvements seen, a key finding from this work is the surprisingly high postdischarge mortality rates in EGS care when studied at 1 year after admission. For those aged over 75 years, the 1‐year mortality rate associated with an EGS admission was more than 33 per cent. In addition, on subgroup analysis, the highly co‐morbid group had a 1‐ year mortality rate above 50 per cent in 2015. Patients who are elderly and have underlying co‐morbidity still have a very high postdischarge mortality rate, which could indicate that EGS admission alone selects for a high‐risk group of patients. EGS admission in these patient groups may provide an opportunity for optimization of medical management, or a joint surgical–geriatric model of care^29^, ^30^, ^31^, ^32^. Furthermore, these outcome data should be in the minds of surgeons when discussing treatment options with patients, particularly frail, elderly individuals^29^, ^33^, ^34^, ^35^.

This study has limitations. The analysis was conducted by admission episode rather than at patient level. Patients admitted to EGS services on several occasions during the last months of their life will be overanalysed in this manner. However, the mortality analysis by episode is well established and forms the basis of indices such as the Summary Hospital Mortality Index. Corrections for multiple admissions are methodologically complex and likely to alter the mortality rates by only a small amount^13^. In addition, studies such as this rely on accurate and consistent coding. The ISD of the NHS Scotland has consistently used professional coders to abstract data, and the quality of the coding has been verified in a number of audits^19^. Lastly, information on the cause of death for each patient during this study was not available. The issues of high postdischarge mortality raise the question of what these patients die from. This information is not directly available, and would require additional permissions and data linkage. However, this study also has many strengths, the most important being that the data provide information on the long‐term outcome of patients, even after discharge. The ability to track patients and their outcomes over time is a key advantage of the Scottish NHS data.

This 20‐year epidemiological study of EGS admissions in Scotland builds on previous analysis, by examining secular trends in mortality at different time points. It confirms that the mortality rate following EGS is high for many patient groups, particularly the elderly and those with co‐morbidity. Furthermore, medium‐term and postdischarge mortality is substantial: surviving an EGS admission does not imply that patients will survive the next year, whether managed surgically or not. However, outcomes have improved over the past two decades, and more so at the later time points than for inpatient mortality. These findings suggest that there has been a gradual overall improvement in the system of care, including after discharge.

## Supporting information


**Table S1** Crude mortality data: number of deaths in‐hospital, at 30 days, 90 days and 1 year
**Table S2** Results of linear regression analysis over time, stratified by age group
**Table S3** Results of linear regression analysis over time, stratified by operative status
**Table S4** Results of linear regression analysis over time, stratified by Charlson Co‐morbidity Index
