# Supplementary material for: Twenty‐year study of in‐hospital and postdischarge mortality following emergency general surgical admission
Source: BJS Open. 2019 Jul 9;3(5):713–21. doi: 10.1002/bjs5.50187 (PMC6773630; doi:10.1002/bjs5.50187)
Supplement: Supplementary file 1 — Table S1 Crude mortality data: number of deaths in‐hospital, at 30 days, 90 days and 1 year Table S2 Results of linear regression analysis over time, stratified by age group Table S3 Results of linear regression analysis over time, stratified by operative status Table S4 Results of linear regression analysis over time, stratified by Charlson Co‐morbidity Index [file BJS5-3-713-s001.docx]

**BJS5_50187**

# Twenty-year study of in-hospital and postdischarge mortality following emergency general surgical admission

**G. Ramsay, J. M. Wohlgemut and J. O. Jansen**

**Table S1** Crude mortality data: number of deaths in-hospital, at 30 days, 90 days and 1 year

| **Year** | **Number of admissions** | **Number of in-patient deaths** | **(%)** | **Number of deaths within 30 days of admission** | **(%)** | **Number of deaths within 90 days of admission** | **(%)** | **Number of deaths within 1 year of admission** | **(%)** |
| --- | --- | --- | --- | --- | --- | --- | --- | --- | --- |
| **1996** | 47 803 | 1 352 | (2.8) | 2 318 | (4.8) | 3 613 | (7.6) | 6 959 | (14.6) |
| **1997** | 65 033 | 1 718 | (2.6) | 3 116 | (4.8) | 5 063 | (7.8) | 9 142 | (14.1) |
| **1998** | 65 232 | 1 715 | (2.6) | 3 061 | (4.7) | 5 051 | (7.7) | 9 278 | (14.2) |
| **1999** | 65 817 | 1 688 | (2.6) | 3 034 | (4.6) | 5 048 | (7.7) | 9 185 | (14.0) |
| **2000** | 69 774 | 1 630 | (2.3) | 3 020 | (4.3) | 5 048 | (7.2) | 9 605 | (13.8) |
| **2001** | 71 977 | 1 659 | (2.3) | 3 074 | (4.3) | 5 156 | (7.1) | 9 638 | (13.4) |
| **2002** | 71 823 | 1 746 | (2.4) | 3 130 | (4.4) | 5 186 | (7.2) | 9 779 | (13.6) |
| **2003** | 71 278 | 1 484 | (2.1) | 3 085 | (4.3) | 5 241 | (7.4) | 9 820 | (13.8) |
| **2004** | 72 313 | 1 415 | (2.0) | 3 055 | (4.2) | 5 144 | (7.1) | 9 847 | (13.6) |
| **2005** | 70 909 | 1 425 | (2.0) | 3 139 | (4.4) | 5 172 | (7.3) | 9 622 | (13.6) |
| **2006** | 71 073 | 1 154 | (1.6) | 2 696 | (3.8) | 4 548 | (6.4) | 9 136 | (12.9) |
| **2007** | 73 585 | 1 129 | (1.5) | 2 754 | (3.7) | 4 770 | (6.5) | 9 292 | (12.6) |
| **2008** | 76 278 | 1 155 | (1.5) | 2 741 | (3.6) | 4 708 | (6.2) | 9 069 | (11.9) |
| **2009** | 77 861 | 1 138 | (1.5) | 2 615 | (3.4) | 4 491 | (5.8) | 9 017 | (11.6) |
| **2010** | 76 756 | 1 066 | (1.4) | 2 525 | (3.3) | 4 432 | (5.8) | 9 066 | (11.8) |
| **2011** | 79 453 | 1 072 | (1.4) | 2 426 | (3.1) | 4 319 | (5.4) | 8 654 | (10.9) |
| **2012** | 81 568 | 993 | (1.2) | 2 408 | (2.9) | 4 232 | (5.2) | 8 548 | (10.5) |
| **2013** | 81 475 | 908 | (1.1) | 2 294 | (2.8) | 3 988 | (4.9) | 8 356 | (10.3) |
| **2014** | 80 955 | 860 | (1.1) | 2 220 | (2.7) | 3 948 | (4.9) | 8 492 | (10.5) |
| **2015** | 79 333 | 772 | (1.0) | 2 241 | (2.8) | 4 059 | (5.1) | 8 238 | (10.4) |

**Table S2** Results of linear regression analysis over time, stratified by age group

**Table S3** Results of linear regression analysis over time, stratified by operative status

**Table S4** Results of linear regression analysis over time, stratified by Charlson Co-morbidity Index
